# Supplementary material for: The organizational principles of de-differentiated topographic maps in somatosensory cortex
Source: eLife. 2021 May 18;10:e60090. doi: 10.7554/eLife.60090 (PMC8186903; doi:10.7554/eLife.60090)
Supplement: Figure 6—source data 1. — Shown are the measured and calculated frequencies (n) of mislocalizations to fingers other than the stimulated one for younger and older adults. The absolute number (n) of mislocalizations to each neighboring finger (1 st, 2nd, 3rd, and 4th) was calculated separately for each age group and across the five stimulated digits (D1–D5). Based on the summed amount of the mislocalizations (Sum), the proportional distribution as expected by chance was estimated for comparison. The chance distribution was estimated in a proportional manner, because the combination of the frequencies of mislocalizations from all five digits (D1–D5) results in eight 1st, six 2nd, four 3rd, and two 4th neighboring fingers. In younger adults for example, the total number of mislocalizations was 942, out of which 387 were made to the 1st neighbors of the five stimulated digits (D1–D5). For D1 to D5, there are eight 1st neighbors in total, so based on chance, we would expect that 377 mislocalizations would be addressed to the 1st neighboring fingers, which is less compared to the number of observed mislocalizations. [file elife-60090-fig6-data1.docx]

| Number of mislocalizations | | | Age Group | Neighbouring finger to the stimulated finger | | | | |
| --- | --- | --- | --- | --- | --- | --- | --- | --- |
|  |  |  |  | 1st | 2nd | 3rd | 4th | Sum |
| Measured distribution | | | younger | 387 | 285 | 202 | 68 | 942 |
| Distribution expected by chance | | | younger | 377 | 283 | 188 | 94 | 942 |
| Measured distribution | | | older | 521 | 350 | 173 | 69 | 1.113 |
| Distribution expected by chance | | | older | 445 | 334 | 223 | 111 | 1.113 |

| **Figure 6-source data 1. Tactile misclocalizations compared to chance level.** Shown are the measured and calculated frequencies (n) of mislocalizations to fingers other than the stimulated one for younger and older adults. The absolute number (n) of mislocalizations to each neighbouring finger (1st, 2nd, 3rd and 4th) was calculated separately for each age group and across the five stimulated digits (D1-D5). Based on the summed amount of the mislocalizations (Sum), the proportional distribution as expected by chance was estimated for comparison. The chance distribution was estimated in a proportional manner, because the combination of the frequencies of mislocalizations from all five digits (D1-D5) results in eight 1st, six 2nd, four 3rd and two 4th neighbouring fingers. In younger adults for example, the total number of mislocalizations was 942, out of which 387 were made to the 1st neighbours of the five stimulated digits (D1-D5). For D1 to D5, there are eight 1st neighbours in total, so based on chance, we would expect that 377 mislocalizations would be addressed to the 1st neighbouring fingers, which is less compared to the number of observed mislocalizations. |
| --- |
