# Supplementary material for: The organizational principles of de-differentiated topographic maps in somatosensory cortex
Source: eLife. 2021 May 18;10:e60090. doi: 10.7554/eLife.60090 (PMC8186903; doi:10.7554/eLife.60090)
Supplement: Figure 6—source data 2. — Shown are results from five robust ANOVAs computed with the factors age and digit on the relative distribution of mislocalizations (in %). One robust ANOVA, based on the 20% trimmed mean, was computed for each of the five stimulated fingers (D1–D5). For all five fingers (D1–D5), a main effect of digit was observed due to the higher amount of mislocalizations to nearer neighbors compared to more distant neighbors. A significant interaction between age and digit was only found for D2. [file elife-60090-fig6-data2.docx]

| Digit | Predictor | *df* | *F* | *p* |
| --- | --- | --- | --- | --- |
| D1 | digit | 3 | 9.70 | 3*10^-4^ |
|  | age group x digit | 3 | 1.73 | 0.191 |
|  | Error | 22.20 |  |  |
| D2 | digit | 3 | 6.58 | 0.002 |
|  | age group x digit | 3 | 4.84 | 0.010 |
|  | Error | 22.02 |  |  |
| D3 | digit | 3 | 9.28 | 3*10^-4^ |
|  | age group x digit | 3 | 0.63 | 0.601 |
|  | Error | 22.88 |  |  |
| D4 | digit | 3 | 3.95 | 0.021 |
|  | age group x digit | 3 | 1.98 | 0.145 |
|  | Error | 22.80 |  |  |
| D5 | digit | 3 | 7.52 | 0.001 |
|  | age group x digit | 3 | 2.41 | 0.096 |
|  | Error | 20.96 |  |  |

| **Figure 6-source data 2: ANOVA results of finger-specific mislocalizations.** Shown are results from five robust ANOVAs computed with the factors age and digit on the relative distribution of mislocalizations (in %). One robust ANOVA, based on the 20% trimmed mean, was computed for each of the five stimulated fingers (D1-D5). For all five fingers (D1-D5), a main effect of digit was observed due to the higher amount of mislocalizations to nearer neighbours compared to more distant neighbours. A significant interaction between age and digit was only found for D2. |
| --- |
