# Supplementary material for: The organizational principles of de-differentiated topographic maps in somatosensory cortex
Source: eLife. 2021 May 18;10:e60090. doi: 10.7554/eLife.60090 (PMC8186903; doi:10.7554/eLife.60090)
Supplement: Transparent reporting form [file elife-60090-transrepform.pdf]

eLIFE
1st Floor
24 Hills Road
Cambridge CB2 1JP, UK
P 01223 855340
W elifesciences.org
T @elife

eLife's transparent reporting form

We encourage authors to provide detailed information *within their submission* to facilitate the interpretation and replication of experiments. Authors can upload supporting documentation to indicate the use of appropriate reporting guidelines for health-related research (see EQUATOR Network), life science research (see the BioSharing Information Resource), or the ARRIVE guidelines for reporting work involving animal research. Where applicable, authors should refer to any relevant reporting standards documents in this form.

If you have any questions, please consult our Journal Policies and/or contact us: editorial@elifesciences.org.

Sample-size estimation

- You should state whether an appropriate sample size was computed when the study was being designed
- You should state the statistical method of sample size computation and any required assumptions
- If no explicit power analysis was used, you should describe how you decided what sample (replicate) size (number) to use

Please outline where this information can be found within the submission (e.g., sections or figure legends), or explain why this information doesn't apply to your submission:

The number of samples (participants) analyzed was determined to ensure that *p*-values for all statistical tests achieved a probability value of 0.05 or less based on Kalisch et al. 2008 (for sensorimotor performance) and Kalisch et al. 2009 (for topographic shifts). This is detailed in the "participant" section of the "Materials and Methods" section.

Replicates

- You should report how often each experiment was performed
- You should include a definition of biological versus technical replication
- The data obtained should be provided and sufficient information should be provided to indicate the number of independent biological and/or technical replicates
- If you encountered any outliers, you should describe how these were handled
- Criteria for exclusion/inclusion of data should be clearly stated
- High-throughput sequence data should be uploaded before submission, with a private link for reviewers provided (these are available from both GEO and ArrayExpress)

Please outline where this information can be found within the submission (e.g., sections or figure legends), or explain why this information doesn't apply to your submission:

Biological replicates are defined as each unique participant, whereas technical replicates are defined as the number of scans and behavioral tests of the same sample associated with protocols and equipment. The number of analyzed samples (biological replicates) is indicated in each figure legend, and in all figure plots, individual data are shown as scatter plots in addition to means and standard errors. We did not encounter any outliers. Exclusion of participants was necessary for the resting state analyses, because for n=9 participants, physiological data could not

eLife Sciences Publications, Ltd is a limited liability non-profit non-stock corporation incorporated in the State of Delaware, USA, with company number 5030732, and is registered in the UK with company number FC030576 and branch number BR015634 at the address 1st Floor, 24 Hills Road, Cambridge CB2 1JP | August 2014

successfully be acquired due to a loss of the pulse oximeter and/or loosening of the breathing belt during MR scanning. For n=4 participants, we observed severe motion artifacts for the resting state data as reported in the "preprocessing" section of the "Materials and Methods" section. For those participants, who participated in the behavioral experiments but could not successfully be measured via MRI, we report behavioral data only.

eLife Sciences Publications, Ltd is a limited liability non-profit non-stock corporation incorporated in the State of Delaware, USA, with company number 5030732, and is registered in the UK with company number FC030576 and branch number BR015634 at the address 1st Floor, 24 Hills Road, Cambridge CB2 1JP | August 2014

Statistical reporting

- Statistical analysis methods should be described and justified
- Raw data should be presented in figures whenever informative to do so (typically when N per group is less than 10)
- For each experiment, you should identify the statistical tests used, exact values of N, definitions of center, methods of multiple test correction, and dispersion and precision measures (e.g., mean, median, SD, SEM, confidence intervals; and, for the major substantive results, a measure of effect size (e.g., Pearson's r, Cohen's d)
- Report exact p-values wherever possible alongside the summary statistics and 95% confidence intervals. These should be reported for all key questions and not only when the p-value is less than 0.05.

Please outline where this information can be found within the submission (e.g., sections or figure legends), or explain why this information doesn't apply to your submission:

Statistical analysis methods are described and justified in the section “Materials and Methods”. This includes the identification of appropriate statistical tests, methods of multiple test correction, and dispersion and precision measures. All figure plots show means, standard errors, and scatter plots that represent individual data. Confidence intervals and effect sizes (Hedge’s g) are reported for substantial results (see Figure 3). P-values are reported for all major statistical tests (irrespective if significant or not) in the text of the “Results” section.

(For large datasets, or papers with a very large number of statistical tests, you may upload a single table file with tests, Ns, etc., with reference to sections in the manuscript.)

Group allocation

- Indicate how samples were allocated into experimental groups (in the case of clinical studies, please specify allocation to treatment method); if randomization was used, please also state if restricted randomization was applied
- Indicate if masking was used during group allocation, data collection and/or data analysis

Please outline where this information can be found within the submission (e.g., sections or figure legends), or explain why this information doesn't apply to your submission:

Participants were allocated into different groups based on their age (young: 20-30 years, old: > 65 years). All participants are part of the institute’s database and randomly selected but controlled for gender (similar gender distribution in both groups). They were selected based on suitability to conduct 7T-MR studies. Due to the strict exclusion criteria for 7T-MRI measurements at our site (as specified in the “Participant” section of the “Materials and Methods” section), it was particularly challenging to recruit older participants due to the higher frequency of co-morbidities; the recruitment process took 4 years (2016-2020). No masking / blinding was used.

Additional data files (“source data”)

- We encourage you to upload relevant additional data files, such as numerical data that are represented as a graph in a figure, or as a summary table
- Where provided, these should be in the most useful format, and they can be uploaded as “Source data” files linked to a main figure or table

eLife Sciences Publications, Ltd is a limited liability non-profit non-stock corporation incorporated in the State of Delaware, USA, with company number 5030732, and is registered in the UK with company number FC030576 and branch number BR015634 at the address 1st Floor, 24 Hills Road, Cambridge CB2 1JP | August 2014

- Include model definition files including the full list of parameters used
- Include code used for data analysis (e.g., R, MatLab)
- Avoid stating that data files are "available upon request"

Please indicate the figures or tables for which source data files have been provided:

We have provided links to code used for model definition of the pRF analyses (links provided in section "Bayesian pRF modeling"), links to code used for the Fourier-based statistics (links provided in section "Phase-encoded analyses"), and links to the toolbox used for resting state analyses (links provided in section "preprocessing"). Data are provided via the sharing platform Dryad.

eLife Sciences Publications, Ltd is a limited liability non-profit non-stock corporation incorporated in the State of Delaware, USA, with company number 5030732, and is registered in the UK with company number FC030576 and branch number BR015634 at the address 1st Floor, 24 Hills Road, Cambridge CB2 1JP | August 2014
